# Metabolic regulation of dendritic cell activation and immune function during inflammation

**DOI:** 10.3389/fimmu.2023.1140749

**Published:** 2023-03-08

**Authors:** Lili Wu, Ziqi Yan, Yiyang Jiang, Yingyi Chen, Juan Du, Lijia Guo, Junji Xu, Zhenhua Luo, Yi Liu

**Affiliations:** ^1^ Laboratory of Tissue Regeneration and Immunology and Department of Periodontics, Beijing Key Laboratory of Tooth Regeneration and Function Reconstruction, School of Stomatology, Capital Medical University, Beijing, China; ^2^ Immunology Research Center for Oral and Systemic Health, Beijing Friendship Hospital, Capital Medical University, Beijing, China; ^3^ Department of Orthodontics School of Stomatology, Capital Medical University, Beijing, China

**Keywords:** metabolic reprogramming, glycolysis, inflammation, dendritic cells (DC), cellular metabolism

## Abstract

Dendritic cells (DCs) are antigen-presenting cells that bridge innate and adaptive immune responses. Multiple cell types, including DCs, rely on cellular metabolism to determine their fate. DCs substantially alter cellular metabolic pathways during activation, such as oxidative phosphorylation, glycolysis, fatty acid and amino acid metabolism, which have crucial implications for their functionality. In this review, we summarize and discuss recent progress in DC metabolic studies, focusing on how metabolic reprogramming influences DC activation and functionality and the potential metabolic differences among DC subsets. Improving the understanding of the relationship between DC biology and metabolic regulation may provide promising therapeutic targets for immune-mediated inflammatory diseases.

## 1 Introduction

Dendritic cells (DCs) are antigen-presenting cells that coordinate innate and adaptive immune responses (1). DCs not only recognize pathogens and danger signals through pattern recognition receptors (PRRs), activate intracellular cascade signals, and release antimicrobial mediators and inflammatory cytokines to initiate the innate immune response but also take up, process, and present antigens to T lymphocytes, promoting adaptive immune response activation (1, 2).

Cells acquire and use nutrients through metabolism to fulfill their energy and biosynthetic demands for physiological processes (3). Studies have indicated that in human and mouse DCs, DC activation is followed by different metabolic alterations that regulate their survival and immune functions (4–6). Active oxidative phosphorylation (OXPHOS) in mitochondria is related to immature DCs (5), whereas enhanced glycolysis after pathogen sensing can boost immunogenic DC activity (5–7). However, increasing evidence suggests that DC activation involves multiple metabolic pathways, including glycolysis, amino acid, and fatty acid metabolism. In various pathological situations, the regulation of DC metabolism after immunogenic activation is complex, i.e. changes in metabolic pathways, molecular signaling pathways regulating cellular metabolism, as well as metabolites and nutrients may affect the function of DCs (4, 8). In addition, different subsets of DCs exhibit distinct metabolic response specializations (4).

Thus, improving the understanding of the impact of metabolic regulation on quiescent DCs and immunogenic DC activation is important. This review highlights the relationship between metabolic adaptations and functional DCs, particularly in inflammation, to identify new therapeutic prospects for inflammatory and immune diseases.

## 2 Heterogeneity of DC populations

DCs are a heterogeneous group of immune cells found in lymphoid tissues (e.g., the lymph nodes [LNs], spleen, and bone marrow [BM]), as well as in the majority of nonlymphoid tissues (9, 10). Classical DCs (cDCs) can be divided into two major subsets: IRF8-dependent cDC1s and IRF4-dependent cDC2s (9). cDC1s are efficient at cross-presenting antigens to CD8^+^ T cells, and cDC2s specialize in CD4^+^ T-cell activation and cytokine generation (9). By contrast, several non-classical DC subsets play a crucial role in peripheral immune surveillance and the infection response. Plasmacytoid DCs (pDCs) are powerful Type-I interferon makers that play a crucial role in viral defense. The subpopulations also contain monocyte-derived “inflammatory DCs” (infDCs), which have a consequence of inflammation or infection (11). The principal characteristics of DC subsets have been described (9, 12–14), and in this section, we briefly review the current models of DCs development (11, 15).

As DCs are relatively few *in vivo*, several *ex vivo* experimental models have been established to investigate DC biology (**Table 1**). For studying human DC physiology, monocyte-derived DCs (moDCs) (16), generated from circulating monocytes stimulated with granulocyte-macrophage colony-stimulating factor (GM-CSF) and interleukin-4 (IL-4), have become a commonly used model (17–19). In addition, mouse DCs *in vitro* are typically produced from BM induced by GM-CSF and IL-4 (BMDCs). MoDCs and BMDCs are the most functionally similar to immature DCs (20, 21), and they are critical for studying DC metabolism and biology.

**Table 1:** In vitro models of DCs.

DC culture		Culture conditions	Cell composition	Metabolic requirements for development	Limitations
**Mouse (from bone marrow)**	BMDCs	GM-CSF (+IL-4), 5-7 days	DC-like cells + GM-Macs	Glucose uptake, OXPHOS, and FAS;	At least two distinct populations
	FLT3L-DCs	FLT3L (+GM-CSF), 9 days	cDC1-like cells	Glucose uptake, FAO and mitochondrial metabolism; higher mitochondrial mass & Δψm than cDC2;	A mixture of cDC1, cDC2, and pDC;
			cDC2-like cells	Glucose uptake & ROS;	
			pDC-like cells	Glucose uptake;	
	iCD103-DCs	FLT3L + GM-CSF, 16 days	cDC1-like cells	Not reported	Less research
**Human (from blood monocyte)**	moDCs	GM-CSF + IL-4, 6-7 days	moDCs	Cytosolic FAS, mitochondrial metabolism, and OXPHOS;	moDCs originate from a different precursor (monocyte vs CDP)

CDP, common or conventional dendritic cell progenitor; GM-Macs, CD11c^+^MHC-II^+^ Macrophages; Δψm, mitochondrial membrane potential.

## 3 Metabolic demands of DCs in quiescence

**Figure 1:**
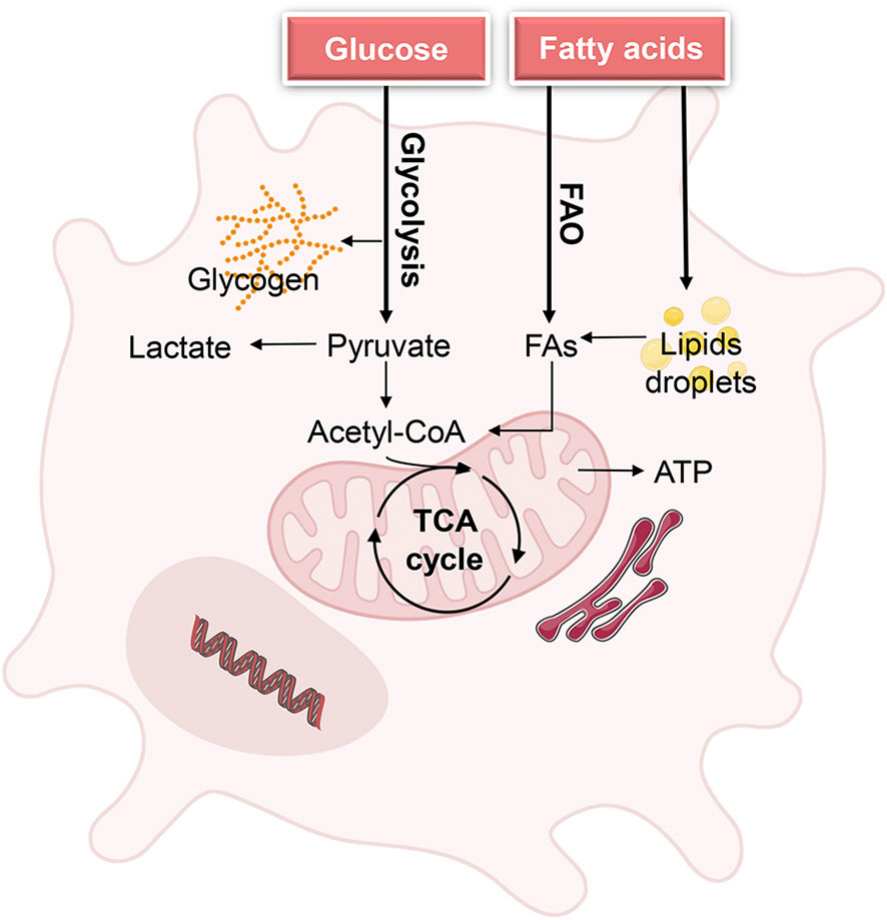
Metabolic demands of DCs during homeostasis. Quiescent DCs in peripheral tissues require glucose and fatty acids as fuels to generate energy for cell maintenance and to build up intracellular glycogen and lipid storage. The metabolic state of quiescence is characterized by active oxidative phosphorylation (OXPHOS), which is driven by the tricarboxylic acid (TCA) cycle Biorender.com.

**Figure 2:**
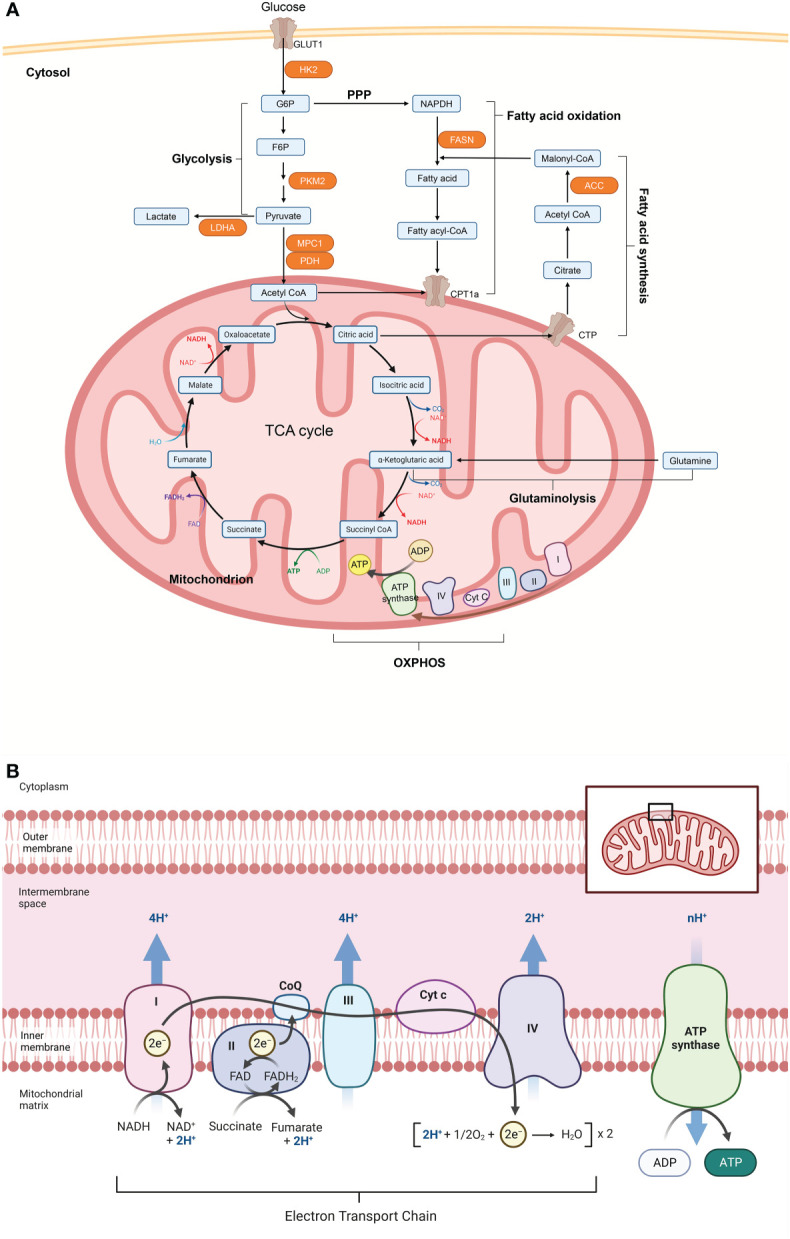
Cellular metabolism networks. **(A)** Glucose is imported from the extracellular environment and can either be converted to glycogen or oxidized during glycolysis to produce adenosine triphosphate (ATP). Pyruvate produced by glycolysis can be partially oxidized to lactate to quickly regenerate the consumed nicotinamide adenine dinucleotide (NADH), or it can be translocated into the mitochondria and completely oxidized *via* the tricarboxylic acid (TCA) cycle. The TCA cycle can also be powered by fatty acids through fatty acid oxidation or glutamine through glutaminolysis. Electrons released by glycolysis and the TCA cycle enter the electron transport chain, which is made up of complex I-V (CI-CV), where ATP is produced through oxidative phosphorylation (OXPHOS). **(B)** Acetyl-CoA from pyruvate enters the TCA cycle in mitochondria. Reactions in the cycle produce NADH and FADH, which act as substrates for the electron transport chain (ETC) and thus support OXPHOS and ATP production. Figure 2 was created with Biorender.com. ACC, acetyl-CoA carboxylase; CoA, coenzyme A; CPT1a, carnitine palmitoyltransferase 1; CTP, citrate transport protein; F6P, fructose 6 phosphate; FASN, fatty acid synthase; G6P, glucose 6 phosphate; GLUT1, glucose transporter 1; HK2, hexokinase 2; LDHA, lactate dehydrogenase A; MPC1, mitochondrial pyruvate carrier 1; NADPH, nicotinamide adenine dinucleotide phosphate; PDH, pyruvate dehydrogenase; PKM2, pyruvate kinase isozyme M2;PPP, pentose phosphate pathway.

During homeostasis, DCs are mostly quiescent in peripheral tissues (**Figure 1**). In resting DCs, glucose is converted to pyruvate through glycolysis (22–24). Some pyruvate is metabolized to lactate, but the majority is sent to the tricarboxylic acid (TCA) cycle *via* acetyl-CoA (24, 25) (**Figure 2**). The mitochondrial electron transport chain receives electron donations from NADH, which is produced by the TCA cycle and predominantly regulated by the Liver Kinase B1 (LKB1)-AMP-activated protein kinase (AMPK) axis (3, 7, 25–27). LKB1 plays a key role in cellular metabolism by controlling AMPK activation (28). LKB1 activates AMPK and AMPK-related kinases, which leads to the upregulation of catabolic pathways and mitochondrial biogenesis while inhibiting anabolic processes (29). Activated AMPK can inhibit mTOR complex 1 (mTORC1) either directly or indirectly *via* its downstream target Tuberous Sclerosis Complex 1 and 2 (TSC1/TSC2) (29). In addition, LKB1 maintains mouse CD11c^+^ DC quiescence in an mTOR-dependent manner, and LKB1 deficiency promotes CD11c^+^ DC activation and metabolic profiles, indicating that LKB1 coordinates immunological and metabolic quiescence in DCs (30). However, in LKB1-deficient CD11c^+^DCs, mTOR inhibition only partially compensated for the loss of LKB1, implying that LKB1 has other targeting pathways to maintain the quiescent state (28, 30, 31).

Furthermore, resting DCs rely on catabolic metabolism to degrade nutrients and generate energy for cell maintenance. The degradation of proteins and triacylglycerols, for instance, yields amino acids (AAs) and fatty acids (FAs), respectively, as fuels for the TCA cycle within mitochondria (7). Additionally, fatty acid oxidation (FAO) was reported to be a key energy metabolic pathway in immature BMDCs, sustaining their survival (7). In steady-state DCs, lipids from the local microenvironment can serve as essential fuel for FAO. Furthermore, resting DCs accumulate glycogen intracellularly, utilize it to fulfill basal glycolytic requirements, and provide metabolic substrates for OXPHOS (32).

## 4 Metabolic reprogramming of immunogenic DCs

When DCs detect changes in the homeostatic state caused by pathogens or tissue-derived inflammatory signals, they shift from the resting state to the active state. Research has shown that metabolic pathways regulate immunogenic DC activation and the subsequent immune responses (6, 19, 33). Here, we discuss the results of recent studies on the metabolic regulation of DC activation, particularly glycolysis. Notably, most of the data on DC metabolism are acquired from DC culture models, namely, BMDCs (mouse systems) (34) and moDCs (human systems) (11).

### 4.1 Critical role of glycolysis in DC metabolic reprogramming

Upon immunogenic activation, DCs frequently convert catabolic metabolism, marked by FAO and mitochondrial respiration, to anabolic metabolism, with increased glycolytic activity and decreased OXPHOS (33). Glycolysis is a key component of glucose metabolism that transforms glucose into pyruvate in the cytoplasm (22–24). The majority of the generated pyruvate transforms into lactate—instead of entering the TCA cycle in the mitochondria—even if oxygen is available. These are classic characteristics of aerobic glycolysis, often known as Warburg metabolism.

#### 4.1.1 Effect of glycolysis on the function of activated DCs

**Figure 3:**
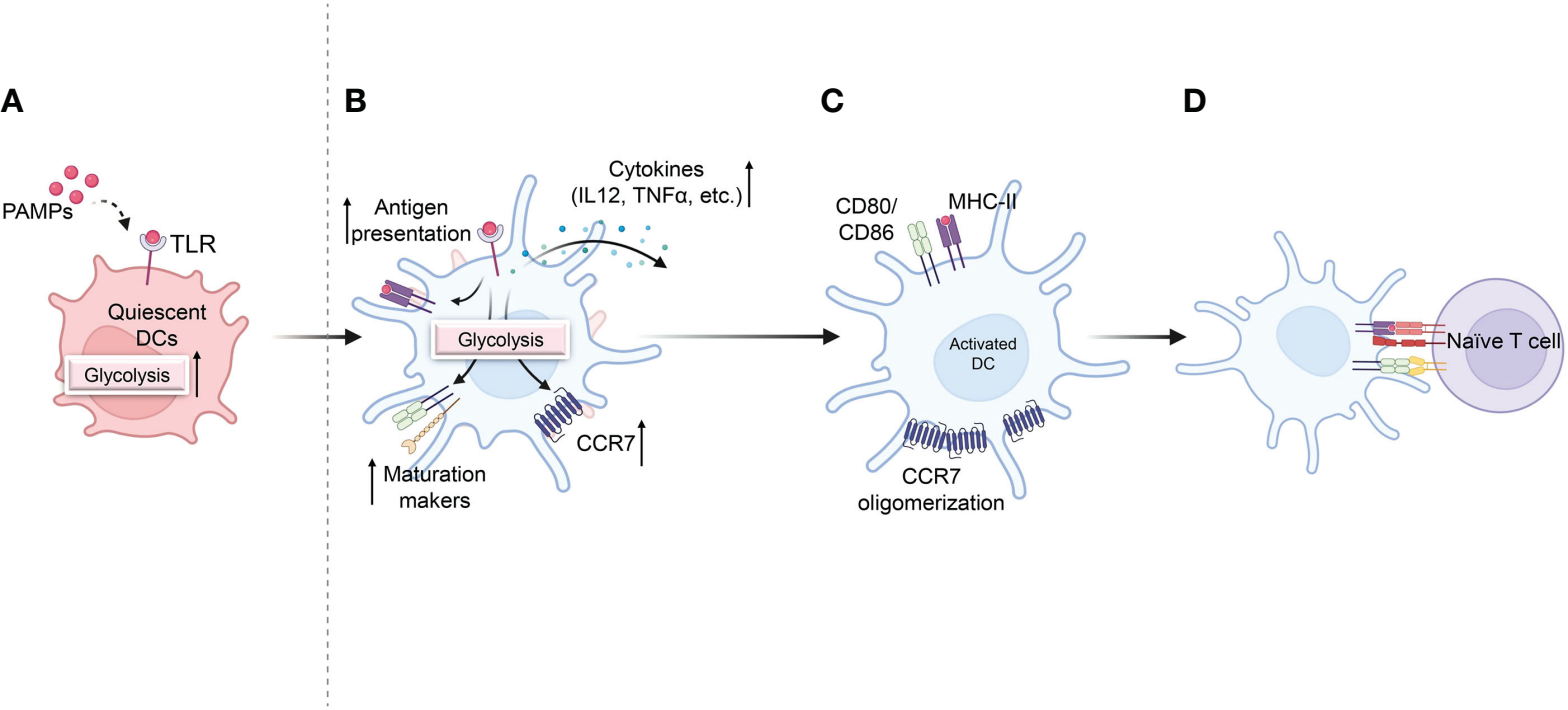
Effect of glycolysis on DC activation and pro-inflammatory activity. **(A)** Quiescent DCs recognize pathogen-associated molecular patterns (PAMPs) that are required for DC maturation, along with a rapid increase in glycolysis. **(B)** Increased glycolysis increases DC activation and antigen presentation after TLR activation. **(C)** Glycolysis also promotes the migration of activated DCs by stimulating CCR7 oligomerization. **(D)** Activated DCs in lymph nodes express co-stimulatory markers, initiating T-cell priming Biorender.com.

Growing evidence suggests that increased glycolysis promotes DC activation and pro-inflammatory function (**Figure 3**), even in different DC cultures and subsets *in/ex vivo* (7, 27, 35, 36). When the methodologies for extracellular acidification and oxygen consumption rates were employed to evaluate BMDC metabolism, stimulation with TLR agonists such as LPS (TLR4) increased glycolytic flux in BMDCs within minutes (7, 37–39). A rapid increase in glycolysis has also been observed in response to LPS activation in moDCs (39). In addition, pharmacological inhibition of glycolysis with 2-deoxyglucose (2-DG) or deficiencies in glycolytic enzymes such as alpha-enolase (ENO1) can significantly impair BMDC maturation and subsequent T-cell activation (7, 36, 40, 41). Similarly, glycolysis is important for DC function *in vivo*. LPS-driven activation of splenic cDC subsets *in vivo* was effectively diminished when mice were given a 2-DG injection to block glycolysis at the same time. Their ability to release IL12 and prime ovalbumin (OVA)-specific CD4 and CD8 T cells in response to LPS was diminished in the presence of 2-DG (41). However, another study found that glucose has a contrasting function in DCs, as it represses the proinflammatory output of LPS-stimulated BMDCs, and negatively affects DC-induced T-cell responses (42). Directly limiting the rate of glycolysis or switching BMDCs from glucose to galactose prevented LPS-stimulated BMDCs from glycolytic reprogramming (42). Furthermore, glucose-deprived BMDCs expressed more costimulatory molecules (CD80, CD86) and IL12 (42), which are known to be important for the induction of T-cell proliferation and the regulation of T-cell effector functions (43).

Furthermore, glycolysis is required for cytoskeletal modifications that allow activated BMDCs to shape and migrate (44). DCs upregulate CCR7 and migrate into lymphatic capillaries, producing CCR7 ligand CCL21 upon TLR activation (**Figure 3**) (45). Suppression of glycolysis impairs dendritic cell shape maintenance, CCR7 oligomerization, and BMDC migration to draining LNs (41, 44). To determine the effect of glycolysis on DC migration *in vivo*, the ability of differentially activated DCs injected in the footpad to migrate to draining LNs was investigated using CFSE-labelled BMDCs (7). BMDCs stimulated with OVA and LPS were more abundant in the LNs than BMDCs stimulated with OVA alone. 2-DG treatment reduced the number of LPS-activated OVA-pulsed DCs in the draining LNs but did not eliminate the effects of LPS (7). Finally, an animal model of allergic asthma induced by HDM was established to monitor DC migration from the lung to the mediastinal LN to investigate the migration of endogenous DCs *in vivo* (46). Administration of 2-DG during allergic inflammation did not significantly impair the accumulation and migration of total immune cells but reduced the migration of endogenous CD11c^+^MHCII^hi^ DCs to the lungs in response to HDM (44). These findings suggest that initiating glycolytic metabolism is critical for full DC maturation and subsequent migration.

Moreover, the effects of glycolysis on DC phagocytosis have not been consistently described. After the BMDCs had been exposed to LPS, hypoxia, or hypoxia and LPS for 24 hours, fluorescently labeled OVA was added to assess the uptake capacity of the differentially treated DC (36). LPS and/or hypoxia decrease the ability of BMDCs to engulf antigens but increase glycolytic activity (36), which has also been reported in moDCs after pathogen-associated molecular patterns (PAMPs) stimulation (47). In the presence of PAMPs, 2-DG activated the inositol-requiring protein 1 (IRE1)/X-box-binding protein 1 (XBP1) arm of the unfolded protein response (UPR) in moDCs, whereas moDCs showed robust phagocytosis as well as a robust ability to release arachidonic acid (47). Nevertheless, a separate study examined the effect of aging on Ag acquisition, processing, and presentation by DCs using a well-established model of cross-presentation (the expression of MHC-peptide on the DC surface). And the study revealed that reduced phagocytic activity in aged mouse splenic cDC1s is associated with mitochondrial dysfunction but not glycolysis (48).

In conclusion, these studies indicate that glycolysis is important for DC activation and pro-inflammatory activity.

#### 4.1.2 Glycolytic reprogramming mechanisms in activated DCs

**Figure 4:**
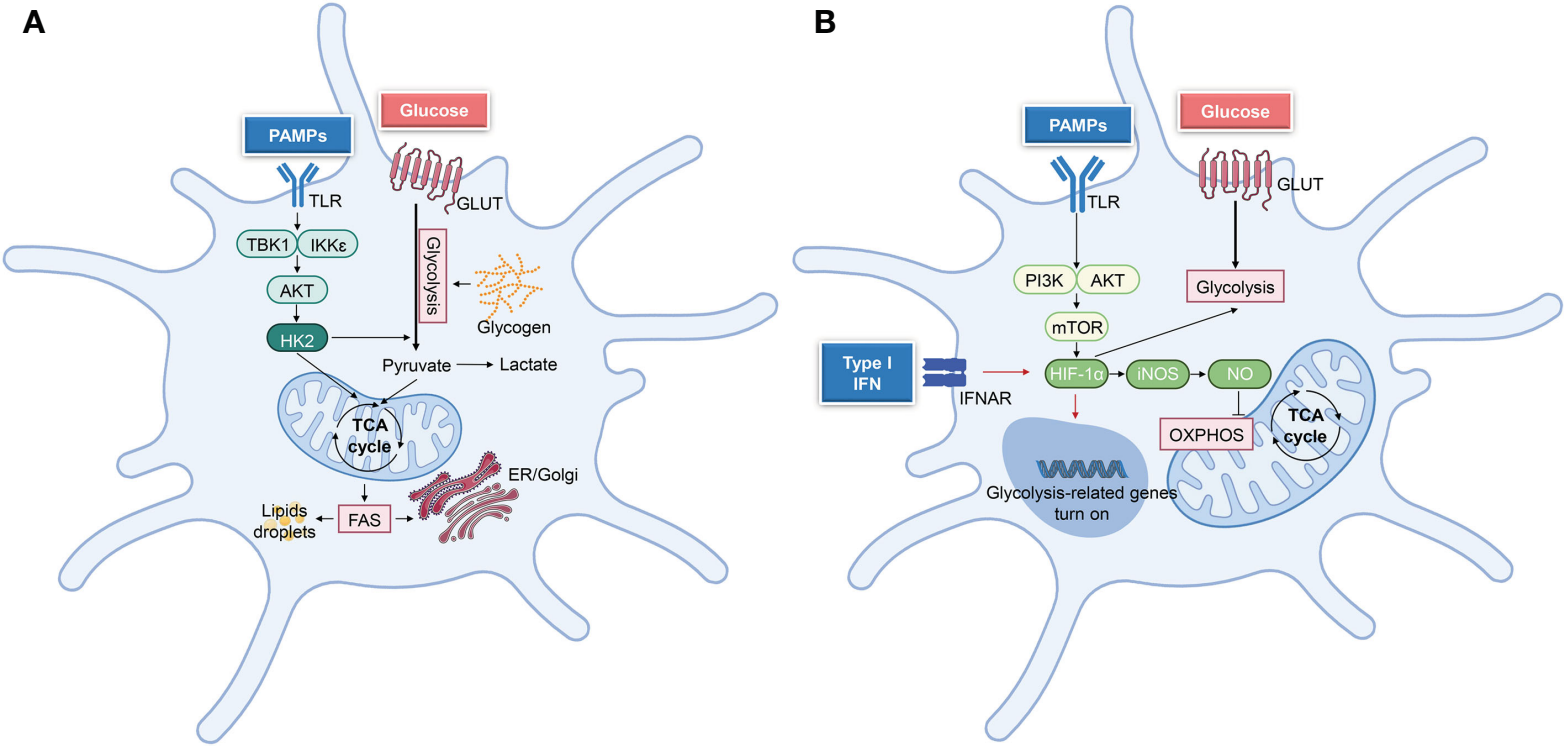
Principal metabolic reprogramming pathways after DCs activation. **(A)** TBK1-IKKε-Akt-HK2 signaling regulates the short-term (minutes to hours following TLR ligation) metabolic regulation in DCs, which undergo rapid metabolic reprogramming. This signaling pathway stimulates glycolysis, which in turn supports other metabolic processes, such as fatty acid synthesis (FAS) and lipid synthesis, hence facilitating DC activation. **(B)** In the later stage of DCs activation, iNOS-dependent glycolysis was performed. TLR ligation triggers PI3K/AKT, and mTOR-HIF1α pathways to promote glycolysis, whereas iNOS-NO suppresses OXPHOS. While activated cDCs do not undergo iNOS-dependent glycolysis *in vivo*, autocrine type-I IFN signaling triggers HIF-1α-mediated glycolytic reprogramming Biorender.com.

DCs undergo two rounds of metabolic reprogramming after activation. These events are triggered by different signaling pathways. TBK1/IKKε/Akt signaling axis mediates early glycolytic reprogramming in BMDCs (**Figure 4**) (41). Within minutes of TLR stimulation, the glycolytic rate of BMDCs doubled and remained elevated for several hours, independent of iNOS signaling (41). It stimulates a non-classical AKT signaling pathway, specifically the TBK1/IKKε pathway, which phosphorylates the glycolysis rate-limiting enzyme, hexokinase 2 (HK2). HK2 then binds to voltage-dependent anion channels on the outer mitochondrial membrane, promoting hexokinase activity (41). These processes improve the glycolytic rate and support early glycolysis induction in LPS-stimulated BMDCs and mouse splenic cDCs (41). Targeted inhibition of TBK1, IKK, or AKT or blocking the binding of HK2 to mitochondria significantly suppressed TLR agonist-induced activation of BMDCs (41). Metabolite tracking experiments have demonstrated that early glycolysis promotes the activation of the pentose phosphate pathway and the preferential production of citrate (41, 49). The citrate generated stimulated fatty acid synthesis (FAS) for endoplasmic reticulum (ER) and Golgi body enlargement to support the increased demand for protein synthesis and transport necessary for BMDC maturation (6, 41, 49, 50). In addition, citrate metabolism promotes the formation of acetyl-CoA, which is required for the epigenetic regulation of glycolytic enzymes such as HK2 (25, 51, 52).

Subsequently, a NO-dependent second wave of glycolytic metabolism is driven by mTOR/HIF1α/iNOS in iNOS-expressing DCs (BMDCs) (**Figure 3**) (38, 53, 54). After LPS stimulation of BMDCs (14 h or more), the PI3K/AKT/mTOR pathway is activated, which upregulates glycolytic enzymes, including phosphofructokinase (PFK), pyruvate kinase 2 (PKM2), and glucose transporters such as GLUT1 (7, 44), which may ultimately increase the extracellular acidification rate (ECAR) (42, 55). In addition, mTOR induces glycolysis through the activation of HIF1α, which upregulates iNOS expression (42, 56) and suppresses NO-mediated mitochondrial activity and OXPHOS in TLR-activated BMDCs (38, 54). Mitochondrial impairment induces a sustained glycolytic metabolism in activated BMDCs to maintain cellular ATP generation and cell growth in the absence of a functional respiratory chain (32, 41, 55). Moreover, iNOS inhibitors can rescue mitochondrial respiration in LPS-activated BMDCs (38).

Unlike BMDCs, the majority of DC subsets, such as natural mouse cDCs and moDCs, do not exhibit detectable levels of iNOS (53). To maintain glycolysis, iNOS-deficient DCs depend on additional mechanisms, such as type-I interferon (IFN) signaling and HIF1α (**Figure 4**). HIF1α contributes to the increased glycolytic activity of mouse cDCs *in vivo*, BMDCs, and moDCs *in vitro* (35, 36, 57). Additionally, intracellular pyruvate or lactate generated by glycolysis can activate HIF1α (58, 59). Activated CD11c^+^MHCII^+^ cDCs promote autocrine IFN-I signaling *via* HIF1α, which decreases mitochondrial OXPHOS levels, increases glycolytic flow, and supplies sufficient ATP for cell activation and survival (57). HIF1α regulates multiple glycolytic genes, including lactate dehydrogenase A (LDHA) and GLUT1, which may be downregulated in DCs once HIF1α loses (36, 42, 47, 60). However, weak activation fails to stabilize HIF1α and induce its target genes, but strong BMDC stimulation results in long-term activation of glycolysis (44). Weak stimulation nevertheless elicits early glycolysis (44), indicating that HIF1α is involved in the maintenance rather than the initial induction of glycolysis (47). However, LPS-activated BMDCs have been shown to exhibit increased IL-12 levels and CD8^+^ T-cell activation in the absence of HIF1α (42). Hence, additional research is required to determine the precise contribution of HIF1α to DC metabolism.

Extracellular glucose and intracellular glycogen serve as the energy source for glycolysis, which is crucial for the survival and function of activated DCs (7, 44, 61). At later stages after LPS stimulation, overexpression of glucose transporters such as GLUT1 increases extracellular glucose absorption (7, 32, 44), whereas inhibition of GLUT1 suppresses the expression of co-stimulatory molecules in BMDCs (31). In addition to the direct utilization of extracellular glucose, intracellular glycogen can be utilized to fulfill the metabolic demands of BMDCs and moDCs through the glycolysis pathway. During the first 6 h after TLRs activation, glycogenolysis of intracellular glycogen stores may sustain enhanced glycolysis more than extracellular glucose (31). Additionally, glycogen phosphorylase inhibitor CP91149 inhibits BMDC maturation and function, particularly during the early stage of activation (31).

### 4.2 Role of fatty acid metabolism and ER stress in the regulation of DC function

FA metabolism is important for the development and function of DCs (62). FAS causes BMDCs to increase lipid storage in lipid bodies (LBs) (41). Increased lipid concentrations in LPS-stimulated BMDCs are closely associated with improved antigen presentation and T-cell activation, suggesting that the *de novo* synthesis of FAs may regulate the immunogenicity of BMDCs (62). Correspondingly, downregulating lipid levels on the membranes of BMDCs through high-density lipoprotein (HDL) and ApoA-I can result in tolerant DCs and reduce T-cell responses (63). Moreover, FAS blockade in LPS-stimulated BMDCs by fatty acid synthase (FASN) or acetyl-CoA carboxylase inhibitors C75 and TOFA, or by the suppression of the mitochondria–cytosol citrate shuttle citrate transport protein, inhibits BMDC activation and pro-inflammatory functions (41).

In LPS-stimulated BMDCs, *de novo* synthesis of FAs is the basis for the Golgi apparatus and ER enlargement (33, 41). CD11c^+^MHCII^+^ DCs isolated from mouse and human livers with high concentrations of lipid increased ER stress and limited their capacity to trigger an immunological response (62). Additionally, PAMP-stimulated moDCs release IL-23 by activating the ER stress response (47). Another study showed that ER stress, characterized by the accumulation of unfolded proteins in the ER lumen, may cause cell death in activated BMDCs (64). However, the UPR can prevent this (64). The UPR coordinates ER expansion and promotes cellular viability by targeting mRNAs encoding XBP1 and IRE1 to increase the synthesis of FAs for ER membranes and proteins that comprise the folding machinery (33, 64).

Lipid accumulation in DCs could be due to increased FAS or increased lipid uptake. Electrospray ionization mass spectroscopy (ESI-MS) analysis of lipid content revealed that BMDCs cultured with tumor explant supernatants (TES) had higher levels of triacylglycerol (TAG), while no changes in the levels of phospholipids and cholesteryl-esters were observed in these DCs (65). The accumulation of oxidized lipids, particularly TAG, can lead to BMDC dysfunction and shorten its lifespan (66). Scavenger receptors (SRs) are an effective route for DCs to acquire fatty acids (67). Experiments with the soluble SR ligand fucoidan and specific antibody to block macrophage scavenger receptor (Msr 1), as well as experiments with Msr1^−/−^ mice, demonstrated that up-regulation of Sra was primarily responsible for increased uptake of exogenous lipids by BMDCs and cDCs (65, 68). When compared to wild-type cells, Msr1-deficient BMDCs displayed a more mature phenotype after LPS stimulation (69, 70), were more responsive to inflammatory stimuli, and had a more effective antigen-presenting capability (69). Fatty acids are most likely transferred to DCs in the form of modified lipoproteins. The molecular mechanisms remain unclear and require further research.

Thus, the regulation of FAS and ER stress, and lipid uptake can affect the function of activated DCs in cytokine release and T-cell activation (27, 33, 65), and further research is required to improve the understanding of the regulation of their metabolic pathways.

### 4.3 Amino acid metabolism in DCs

AAs are involved in various metabolic activities and are crucial for controlling DCs’ function. DCs are vulnerable to environmental changes caused by AA concentrations. In immature moDCs, an imbalance in intracellular AAs impairs mitochondrial activity, decreasing ATP production and increasing glucose uptake, which cannot be further increased by LPS stimulation (71). The plasma of liver cirrhosis patients typically reveals an imbalance between lower levels of branched-chain amino acids (BCAAs) and higher levels of aromatic AAs. moDCs cultured in a medium containing similar AA concentrations showed impaired maturation, IL-12 secretion, and migratory potential after LPS stimulation (71, 72). LPS treatment increased BCAAs uptakes, such as isoleucine, leucine, and valine, in moDCs. BCAAs deficiency, particularly valine deficiency, can inhibit the maturation of moDCs, evidenced by decreased co-stimulated molecular expression (CD40, CD80, CD86, HLA-DR) (71). Another study revealed that BCAAs might regulate human moDC metabolism through the mTOR pathway to affect moDC maturation (73).

It was also demonstrated that LPS-stimulated moDCs increase the absorption of glutamate, cysteine, and aspartate (71). However, blocking the activity of the cystine or glutamate antiporter decreased glutathione synthesis but had no impact on the maturation and antigen uptake of moDCs (74). Additionally, inhibiting glutaminolysis did not affect the metabolic activities that occurred 6 h after LPS treatment, and reducing glutamine in BMDCs culture media (from 2 to 0 mM) did not affect co-stimulated molecular expression (32, 41). However, treatment with l-homocysteine acid (LHC), a glutathione production inhibitor, impairs the ability of murine spleen CD11c ^+^DCs to present antigens to CD4^+^ and CD8 ^+^ T lymphocytes (74).

The role of AAs in DC activation has not been elucidated, and further research is required to determine the significance of various AAs in DC metabolism.

### 4.4 Limitations of *in vitro*-generated DCs for studying cellular metabolism

DCs are extremely rare in tissues (<2%), and the isolated procedures are complex, necessitating lengthy enzymatic digestion steps that may affect their phenotype and activity. Furthermore, once cultured ex vivo, they are vulnerable to spontaneous activation and cell death (75, 76). Hence, the majority of the data on DC metabolism are based on the use of *in vitro*-generated DCs. There are several differences in cellular metabolic programs between *in vitro*-generated DC models and ex vivo primary DC subsets (**Tables 1**, **2**).

**Table 2:** Metabolic regulation of DC subsets in vivo.

DC subsets	Developmental origin	Main locations	Metabolic requirements in vivo for development and main signaling factors
cDC1s	HSC→CDP→pre-cDCs	Lymphoid-resident, peripheral tissue, blood	M: Reduced upon energy restriction; more reliant on OXPHOS and functional mitochondrial metabolism than cDC2 or pDCs; LKB1-AMPK-mTOR axis, Mst1/2 H: Mst1/2
cDC2s			M: Reduced upon energy restriction; LKB1-AMPK-mTOR axis H: Not reported
pDCs	HSC→CDP→pre-pDCs	Lymphoid-resident, blood	M: mTORC1, TSC1 H: mTORC1, PI3K, PKB
infDCs	HSC→CMP→monocyte	Mainly induced upon inflammation in peripheral tissue	Not reported

HSC, hematopoietic stem cell; M, mouse; H, human.

The most widely used *in vitro* DC models are BMDCs. Notably, BMDCs in culture are heterogeneous and contain a population of CD11c^+^MHC-II^+^ Macrophages (GM-Macs) (34). In contrast with BMDCs, GM Macs are mainly immobile and release high quantities of inflammatory cytokines and chemokines in response to microbial stimulation (34). BMDCs and GM Macs have distinct functional properties, which means that their metabolic requirements may be different. Notably, differences in culture conditions can change the ratio of cultured BMDCs to GM-Macs, which may result in different experimental results in the field of cellular metabolism. Furthermore, activated GM-Macs express iNOS, which suppresses mitochondrial activity and OXPHOS by producing NO (77, 78), changing the cellular metabolic machinery. After LPS activation, an increase in glycolysis is caused by NO-induced inhibition of OXPHOS in LPS-stimulated BMDCs cultures (7, 38). Therefore, using a BMDCs model to study DC metabolism is not ideal.

Later, a protocol that relies on the addition of Flt3L to bone marrow progenitors was developed (79). Flt3L is the main growth factor driving DC differentiation, and its addition results in DC subsets similar to those found in the spleen under resting conditions. However, the culture of hematopoietic progenitors with Flt3L generates a mixture of cDC1, cDC2, and pDCs (79, 80). If the study of a single population is desired, additional purification steps may be required. A variation of the original Flt3L-DC culture that co-culture with OP9 stromal cells expressing the Notch ligand Delta-like 1 (OP9-DL1), was recently proposed (80). The authors supporting this modified protocol claim that the addition of the Notch ligand DL1 can induce IRF8-dependent cDC1s with a phenotype and expression profile similar to primary splenic cDC1s (80). Besides, another protocol showed that bone marrow cells cultured in the presence of Flt3L and GM-CSF produced a high number of CD103^+^ cDC1s (81). However, few metabolic studies have been performed based on these DC cultures.

moDCs, generated from GM-CSF+IL-4 cultured blood mononuclear cells, is the most commonly used model for human DC studies *in vitro* (16). Despite several functional and phenotypic similarities with the human DC population found *in vivo*, moDCs are derived from a different precursor cell monocyte (cDC-CDP) and have a low migratory capacity *in vivo*. In addition, transcriptome research has revealed that human moDCs are more strongly linked to monocytes and macrophages than blood DCs are (82).

DCs generated *in vitro* can be produced quickly in large quantities and are particularly useful in the field of cell metabolism, in which high cell counts are necessary for experiments (27); however, they have limitations. In further research, new models closer to the *in vivo* situation are required to study the metabolic requirements of DCs.

## 5 Cellular metabolism of cDC and pDC subsets

As described, cellular metabolic programs can influence DC function. However, early metabolic investigations using *in vitro* BMDCs and moDCs cultures failed to correspond with natural DCs subsets. Next, we discuss the research in the field of metabolism of different mouse and human DCs subsets, focusing on the differences between cultured models (**Table 2**). The combination of these two aspects may contribute to the progress in understanding DC metabolism and deepen the physiological understanding, which is critical for developing effective DC-based therapeutics.

### 5.1 Metabolic features during DCs development and differentiation

Little is known about the metabolic pathways that support multiple stages of differentiation of BM progenitors to fully differentiated DCs. DC subpopulations were generated with different energy requirements for different functions (**Table 1**).

The LKB1-AMPK-mTOR axis plays a crucial role in maintaining DC quiescence and can be activated during DC differentiation. Studies have shown that AMPK and LKB1 play important roles in DC differentiation. A reduced proportion of cDC1 was observed in the LN of AMPKa gene-deficient mice, suggesting a dominant role for AMPK in terminal cDC1 differentiation (83). A similar effect has been observed in conditional gene-deficient mice with LKB1-deficient CD11c^+^ cDCs, with a higher proportion of cDC2 in the mouse thymus (28, 30). The LKB1-AMPK axis helps determine cell differentiation during DC development, and deletion of either molecule is detrimental to IRF8^+^ cDC1 differentiation (83). Notably, cDC1 and cDC2 have been shown to have extremely pronounced metabolic differences at baseline, where splenic cDC1 appears to be more reliant on OXPHOS and functional mitochondrial metabolism than cDC2 or pDCs (48, 83).

Indeed, *in vitro* and *in vivo*, cDC1 had a larger mitochondrial mass and mitochondrial membrane potential (ΔΨm) than cDC2s (48, 83, 84), consistent with higher AMPK activity and increased oxidative metabolism (27). Inhibiting catabolic processes such as AMPK signaling, FAO, or mitochondrial clearance did not affect total cDC/pDC development but significantly increased the frequency of IRF4^+^ cDC2 cells while decreasing the frequency of IRF8^+^ cDC1 cells. Scavenging anabolism-associated reactive oxygen species (ROS), by contrast, tilted differentiation toward cDC1 cells (83). In addition, a data-driven systems biology algorithm (NetBID) study revealed significant enrichment of Mst1 and Mst2 (Mst1/2) activity, the non-canonical Hippo pathway kinases, in cDC1 cells relative to cDC2 cells. Mechanistically, cDC1 has a substantially greater oxidative metabolism than cDC2 and relies heavily on Mst1/2 signaling to maintain metabolic activity and mitochondrial integrity for immunogenic function (84). However, in the steady state, CD11c-Cre Mst1/2^flox/flox^ mice have increased splenic cDC1 frequencies, unaffected pDCs, and decreased cDC2s (84), implying that understanding the precise role of Hippo/Mst signaling in DC formation requires further exploration.

### 5.2 Regulation of energy metabolism by DCs activation - specific to cDCs and pDCs subpopulations

Information on the metabolic pathways involved in cDCs activation *in vivo* is limited. Next, we briefly outline the literature on cDC subsets in the metabolic field. Splenic mouse cDC1 and cDC2 enhance their ECAR quickly after *in vivo* LPS stimulation (41); nevertheless, after *ex vivo* LPS stimulation for 24 h, they do not differ in their ECAR/OCR ratio (38). *In vivo* stimulation with poly(I: C) decreased the OCR and ΔΨm of total spleen cDCs, which was blocked by IFNAR elimination (57). TLR activation decreases the mitochondrial content, increases OXPHOS activity, and stimulates glycolysis in human blood cDC2. TLR-stimulated glycolysis and cDC2 activation are impaired when mitochondrial fragmentation is inhibited or when mitochondrial fusion is promoted. TLR stimulation induces BNIP3-dependent mitophagy, which is essential for glycolysis induction and cDC2 activation (85).

The understanding of metabolic reprogramming in pDCs is relatively restricted, but recently, studies have demonstrated that pDCs exhibit differential rewiring of their mitochondrial energy metabolism in different environments. After *ex vivo* infection with influenza or rhinovirus, OCR decreases in human pDCs (86), whereas TLR7/8 stimulation boosts their glutaminolysis and OXPHOS (85). TLR7/8-stimulated pDC activation requires autophagy-supplemented glutaminolysis to fuel OXPHOS, which is necessary for CD80 and IFNα expression (85). In addition, mouse pDCs isolated from FLT3L-DC culture exhibited enhanced glycolytic flux and OXPHOS approximately 24 h after TLR9 stimulation. Increased FAO of *de novo*-produced fatty acids drives an increase in mitochondrial metabolism. This impact is the result of autocrine or paracrine type I IFNs, with IFNα regulating FAO in pDCs (87). TLR-induced pDC activation is suppressed by pharmaceuticals that inhibit FAS or block the action of Cpt1a (87, 88). TLR3 stimulation increases mitochondrial-derived ROS in pDC, allowing them to stimulate CD8 ^+^ T-cell responses *via* cross-presentation (89).

Additionally, activated pDCs consumed more glucose than unstimulated pDCs with increased ECAR. pDC activation is linked to alterations in glycolysis and mTORC1 activity (90). The presence of appropriate amounts of AAs in the environment is required for mTORC1 activation, with leucine and methionine being particularly important (91, 92). Induction of the system L amino acid transporters SLC7A5 and SLC3A2 in pDCs, as well as leucine uptake mediated by these transporters, is required for priming future mTORC1 activation and cytokine production by activated pDCs (90). These results demonstrate that the coordinated actions of mitochondria, glycolysis and fatty acid metabolism are essential for pDC function.

## 6 Conclusions and future perspectives

Although new knowledge on the metabolic control of DCs has recently been revealed, many intriguing questions remain unresolved because the exploration of DC metabolism is nascent.

Current investigations on the impact of the metabolic microenvironment on DCs are primarily conducted with BMDCs (murine) or moDCs (human) *in vitro* and generally involve alterations to only a single gene or metabolite. Although these studies have provided a foundation for understanding the metabolic regulation of DC function, culture conditions *in vitro* frequently do not accurately reflect the complexity of different metabolites *in situ in vivo*. DCs are commonly cultured in DMEM and RPMI 1640 with higher levels of glucose and lower levels of electrolytes, such as magnesium and calcium (93). Furthermore, whether various DC subsets have different metabolic demands or whether their functions depend on similar metabolic programs remains unclear. The effects of other metabolic processes, including the pentose phosphate pathway and nitrogen metabolism pathways, on DC differentiation remain unknown. Moreover, little is known about the cross-talk between metabolic pathways and other epigenetic or molecular regulatory pathways, such as microRNAs, cytokines, and transcription factors, for determining the function or differentiation of DCs.

The metabolic microenvironment has a substantial impact on DC function and dysregulated DC metabolism can contribute to various diseases, including cancers (94, 95), autoimmune diseases (96), and inflammatory disorders (97, 98).

Consequently, reprogramming the metabolic status of DCs could be an efficient means of regulating inflammation. For example, limiting glucose and fat uptake could decrease their pro-inflammatory effects on tissue-associated DCs and subsequently reduce diabetes-associated inflammation (99–101). Additionally, approaches that directly target critical regulators of certain metabolic pathways, such as AMPK, mTOR, or the addition of specific nutrients, restrict the susceptibility of DCs to extracellular environment alterations and hence control inflammation (55, 102).

In conclusion, understanding how DC immunological activity is regulated by metabolism is essential. Using metabolic modulation will advance the understanding of DC biology and immune regulation in inflammatory diseases and facilitate the exploration of effective DC-based immunotherapies.

## Author contributions

LW contributed to the design and conception, the search of the literature, the creation of figures, drafting and critically revising the manuscript; ZY and YJ contributed to the search of the literature and critically revising the manuscript; YC contributed to the creation of figures and revising the manuscript; JD, LG, and JX contributed to the revision of the manuscript; ZL and YL contributed to the conception, design, and critically revising the manuscript. All authors contributed to the article and approved the submitted version.
